# Genome-wide association study of alcohol dependence in male Han Chinese and cross-ethnic polygenic risk score comparison

**DOI:** 10.1038/s41398-019-0586-3

**Published:** 2019-10-07

**Authors:** Yan Sun, Suhua Chang, Fan Wang, Hongqiang Sun, Zhaojun Ni, Weihua Yue, Hang Zhou, Joel Gelernter, Robert T. Malison, Rasmon Kalayasiri, Ping Wu, Lin Lu, Jie Shi

**Affiliations:** 1 National Institute on Drug Dependence, Peking University, 100191 Beijing, China; 2 Peking University Sixth Hospital, Peking University Institute of Mental Health, NHC Key Laboratory of Mental Health (Peking University), National Clinical Research Center for Mental Disorders (Peking University Sixth Hospital), Peking University, 100191 Beijing, China; 3 Beijing Hui Long Guan Hospital, 100096 Beijing, China; 4 The Second Affiliated Hospital, Xinjiang Medical University, 830063 Urumqi, China; 5 Department of Psychiatry, Yale University School of Medicine, New Haven, CT 06511 USA; 6 VA Connecticut Healthcare System, West Haven, CT 06516 USA; 7 Clinical Neuroscience Research Unit, Connecticut Mental Health Center, New Haven, CT 06519 USA; 8 Department of Psychiatry, King Chulalongkorn Memorial Hospital, Bangkok, 10330 Thailand; 9 Department of Psychiatry, Faculty of Medicine, Chulalongkorn University, Bangkok, 10330 Thailand; 10 Beijing Key Laboratory of Drug Dependence Research, Peking University, 100191 Beijing, China; 11 The State Key Laboratory of Natural and Biomimetic Drugs, Peking University, Beijing, 100191 Beijing, China; 12 The Key Laboratory for Neuroscience of the Ministry of Education and Health, Peking University, 100191 Beijing, China

**Keywords:** Comparative genomics, Addiction

## Abstract

Alcohol-related behaviors are moderately heritable and have ethnic-specific characteristics. At present, genetic studies for alcohol dependence (AD) in Chinese populations are underrepresented. We are the first to conduct a genome-wide association study (GWAS) for AD using 533 male alcoholics and 2848 controls of Han Chinese ethnicity and replicate our findings in 146 male alcoholics and 200 male controls. We then assessed genetic effects on AD characteristics (drinking volume/age onset/Michigan Alcoholism Screening Test (MAST)/Barratt Impulsiveness Scale (BIS-11)), and compared the polygenic risk of AD in Han Chinese with other populations (Thai, European American and African American). We found and validated two significant loci, one located in 4q23, with lead SNP rs2075633*ADH1B (*P*_discovery_ = 6.64 × 10^−16^) and functional SNP rs1229984*ADH1B (*P*_discovery_ = 3.93 × 10^−13^); and the other located in 12q24.12-12q24.13, with lead SNP rs11066001*BRAP (*P*_discovery_ = 1.63 × 10^−9^) and functional SNP rs671*ALDH2 (*P*_discovery_ = 3.44 × 10^−9^). *ADH1B* rs1229984 was associated with MAST, BIS_total score and average drinking volume. Polygenic risk scores from the Thai AD and European American AD GWAS were significantly associated with AD in Han Chinese, which were entirely due to the top two loci, however there was no significant prediction from African Americans. This is the first case-control AD GWAS in Han Chinese. Our findings demonstrate that these variants, which were highly linked with *ALDH2* rs671 and *ADH1B* rs1229984, were significant modulators for AD in our Han Chinese cohort. A larger replication cohort is still needed to validate our findings.

## Introduction

Alcohol dependence (AD) is a common medical and social issue. The harmful effects of alcohol include chronic disease and injury and is a serious and growing problem worldwide^1^. In China, the current prevalence of AD in adult males is about 4.54%^2^. Compared to other countries, China has a different demographic profile and a specific “drinking culture”. Population-specific research regarding AD is essential for the basic understanding of the phenotype, and for the development and assessment of national policies for alcoholism prevention.

Although environmental variables such as patterns of alcohol consumption can influence the development and severity of AD, twin and family-based studies have consistently demonstrated that the heritability of AD is ~50%^3^. Identifying genetic risk factors for AD could extend our understanding of the biological mechanisms and would be helpful for individualized prevention and control of alcohol-related diseases. The genetic susceptibility to alcohol dependence has been widely explored using genome-wide association studies (GWAS) and candidate-gene studies across different populations^4–6^. GWAS has demonstrated the polygenic nature of AD. Alcohol is sequentially metabolized by alcohol dehydrogenases (ADH) and aldehyde dehydrogenases (ALDH); the most reproducibly identified risk variants for AD and AD-related traits have been mapped to alcohol metabolizing enzyme genes, particularly located in *ADH1B* and *ALDH2*^7–11^. Impulsivity is clearly related with alcohol use, and was consistently found to be elevated in alcoholics and heavy drinkers^12^. Hence, risk effects of AD-related variants on impulsivity are of interest^13,14^.

Genetic factors related to AD vary across different populations. East Asians have a certain amount of high-activity ADH variants at higher minor allele frequency (MAF) compared to other populations. Inactivating ALDH variants could promote acetaldehyde accumulation and could be protective against heavy drinking and AD^15^. However, published genome-wide data regarding AD in Chinese populations are rare^16,17^, with only one published case-control AD GWAS (102 cases and 212 controls) conducted in AD extended pedigrees^10^.

We conducted a GWAS with 533 unrelated Han Chinese AD patients and 2848 controls in the discovery stage, then validated our results using an independent cohort of 146 AD patients and 200 controls. We then assessed the genetic effects of the target SNPs on AD measures and impulsivity traits. In addition, the genetic overlap of AD genetic risks between Han Chinese and other populations (Thai, European American and African American) were investigated using polygenic risk score analysis.

## Methods

### Subjects

A total of 653 male AD inpatients were recruited from 12 psychiatric hospitals in northern China (Beijing, Inner Mongolia Autonomous Region, Shandong, Tianjin, Jilin, Liaoling and Heilongjiang provinces). All patients sought treatment for AD and were clinically determined based on the Diagnostic and Statistical Manual of Mental Disorders 4th edition (DSM-IV)^18^ by experienced psychiatrists. They had not used any other addictive substances except for nicotine based on their self-reports. The included participants were assessed using the MINI-International Neuropsychiatric Interview (M.I.N.I.) for other current psychiatry disorders. AD patients who smoked were assessed using the Fagerström Test for Nicotine Dependence (FTND). The 2854 healthy controls (HCs) were recruited from local communities through advertisements and broadcasting in neighborhood committees. All HCs were exposed to alcohol (confirmed by the “yes” answer to the question “have you ever consumed alcohol in your life?”) but were not diagnosed as AD. In addition, they lacked a history of drug abuse (except nicotine) and psychiatric disorders according to their self-reports. Healthy controls were not assessed for their education level, alcohol and nicotine use.

The replication cohort included 146 male AD inpatients and 200 male HCs recruited from Peking University Sixth Hospital and four other psychiatric hospitals (Anhui, Shangdong, and Henan provinces) and local communities in these cities. This was through advertisements and broadcasting in neighborhood committees. The inclusion and exclusion criteria were similar to the discovery cohort.

All subjects were at least 18-years-old and provided written informed consent prior to their study participation and were compensated for their participation. The Institutional Review Board of Peking University Health Science Center approved the study protocol.

### MAST and BIS tests and alcohol use characteristics

Among the patients in the discovery cohort, AD severity of the 435 patients were measured using criterion counts of the Chinese version of the Michigan Alcoholism Screening Test (MAST)^19^, a 22-item self-scoring test. MAST had high sensitivity and specificity for lifetime AD, and performed well with the DSM-IV criteria^20^.

In addition, we used the Barratt Impulsiveness Scale 11th version (BIS-11) to assess impulsivity. The BIS-11 is a 30-item self-report questionnaire that assesses impulsiveness for three factors (attentional, motor and non-planning) and the sum score of these three factors was the BIS_total score. Subjects were administered the Chinese version of the BIS-11^21^. Self-reported alcohol characteristics in AD patients including age of onset of regular alcohol consumption, AD duration, average quantity of daily alcohol consumption (the usual daily drink volume), and the maximum drink volume per day (maximum daily drink volume). The number of drinks were coded as standard drinks. To adjust for multiple testing, we applied the threshold of Bonferroni correction (*P* < 0.05/6).

### Genotyping and quality control

Genotyping for the discovery cohort was performed using the Illumina Global Screening Array-24 v1.0 BeadChip (Illumina, Inc., San Diego, CA, USA). Among the 653 cases, 571 had suitable DNA for genotyping. Of the total 700,078 SNPs, 293,054 SNPs with minor allele frequency (MAF) <0.01 and 21,867 SNPs with call rate <95% were excluded; 13 case samples were removed due to the threshold of call rate <95%; 25 case samples and 6 controls were removed due to autosomal heterozygosity >5 s.d. away from the mean or being one of a pair of individuals with proportion identity by descent (IBD) PI_HAT ≥0.185 (the one with the lower call rate was excluded). After quality control, 2164 SNPs were further excluded due to call rate <95%, MAF < 0.01 or Hardy–Weinberg (H–W) equilibrium test *P* < 1 × 10^−6^. The final dataset included 533 cases and 2848 controls with 382,993 SNPs.

A total of 20,141 genotyped autosomal SNPs which were in low linkage disequilibrium (LD) (MAF > 0.35 and *r*^*2*^ < 0.05 for each pair of SNPs) and absent from the 5 long-range LD regions^22^ were included for principal component analysis (PCA) using EIGENSOFT 4.2 software^23,24^. The top 10 principal components (PCs) were used as covariates in subsequent association analysis.

We genotyped two lead SNPs (rs2075633 and rs11066001) and two previously implicated functional SNPs (rs1229984 and rs671) for the two significant loci in the replication cohort (146 cases and 200 controls) using the Agena MassArray Analyzer 4.0 (Agena Bioscience, Inc., San Diego, CA, USA). The significant *p* level was set as *P* < 0.05 for genetic association in the replication cohort.

### Imputation

Genotype imputation was performed using the pre-phasing/imputation stepwise approach implemented in IMPUTE2^25^ and SHAPEIT^26^. The imputation reference set consisted of 2186 phased haplotypes from the full 1000 Genomes Project Integrated Phase 1 Release (March 2012)^27^. Imputed SNPs with info <0.6 or SNPs with minor allele frequency <0.01 were removed. After quality control and imputation, a total of 6,449,738 SNPs for 533 cases and 2848 controls were included for the discovery GWAS.

### Statistical analysis

The imputed data was used for the association analysis in the discovery stage using SNPTEST^28^ (-frequentist 1, -method score) (https://mathgen.stats.ox.ac.uk/genetics_software/snptest/snptest.html). Gender, age and top 10 PCs were used as covariates. Association analysis for the replication stage was performed in PLINK 1.9^29^ using an additive model in logistic regression with age as covariates. The sample size weighted method in METAL^30^ was used for meta-analysis of the two stages. All association test *p*-values were two-sided and were reported without correction for multiple testing. Genome-wide significance was considered as *P* < 5 × 10^−8^. SNP functional annotation was performed using Annovar^31^. Mediation analysis was performed using the model 4 in PROCESS^32^. Here, we mainly focused to bootstrap the sampling distribution of the indirect effect of significant SNPs (X) on AD severity (Y) which was mediated by the BIS_total score (M), or indirect effect of significant SNPs (X) on impulsivity (Y) through the mediation of AD severity (M). Age and the top 10 PCs for the genetic association analysis were used as covariates. *N* = 10,000 was used for bootstrap. Sobel test was further used to show the *P*-value which denotes if the indirect effect was different with zero.

### Polygenic risk score analysis

Polygenic risk scores (PRS) derived from GWAS summary statistics of the same or related traits from other datasets could be used to test the genetic relationship of those traits with the study trait^33^. PRS was calculated as the sum of the risk alleles with p-values less than the threshold of significance. GWAS summary data for AD from the Thai population was obtained by meta-analyzing the two samples (242 cases and 268 controls genotyped using the Illumina (San Diego, CA, USA) Multi-Ethnic Global Array (MEGA), 129 cases and 406 controls genotyped using Illumina Global Screening Array (GSA))^7^. We also included the AD GWAS summary statistics for European-Americans (EA) and African-Americans (AA) from the latest PGC alcohol dependence GWAS (*n* = 4061)^34^. We derived PRS using six *p*-value thresholds (5 × 10^−5^, 2 × 10^−4^, 5 × 10^−4^, 0.001, 0.01, 0.1). The associations between the derived PRS and the phenotype (AD vs. controls) were tested using a logistic regression model, adjusted for age, gender, and the top 10 PCs. Prior to PRS analysis, we obtained the plink format of the imputated discovery data using gtool. PRS analysis was then performed using PRSice-2^35^. Default parameters were used, including using OR as effect size estimates (‘-stat OR’), using additive model (‘--model add’) for regression, taking the average effect size (‘-score avg’) to calculate the polygenic score, using ‘-clump-kb 250, -clump-r2 0.1 –clump-p 1’ for LD clumping. We performed gene and gene-set association analysis using MAGMA^36^, and applied Bonferroni correction for multiple testing.

## Results

### Chinese AD case-control GWAS

**Fig. 1:**
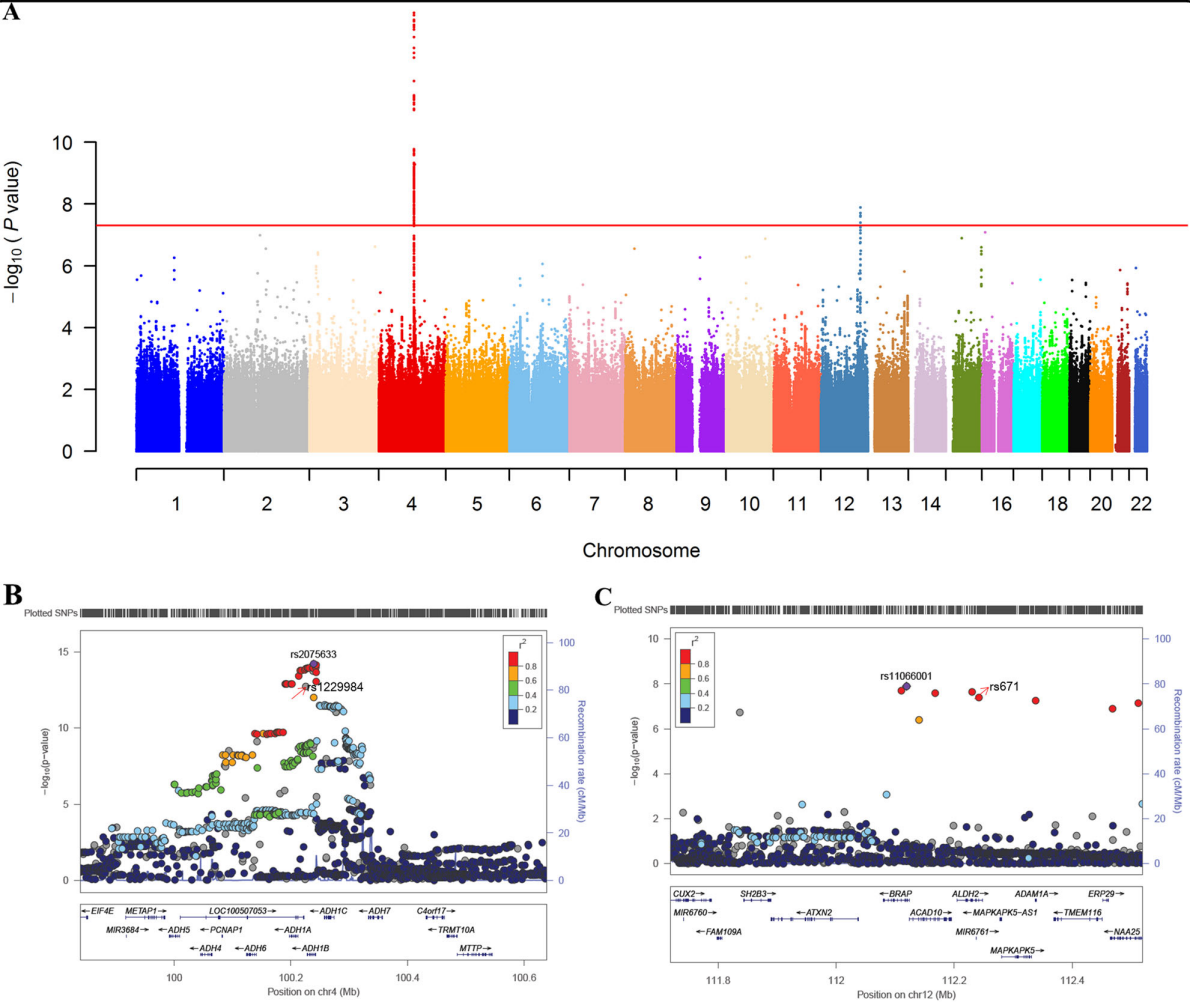
Significant loci associated with alcohol dependence in Han Chinese. **a** Manhattan plot for the discovery cohort. Red line denotes the threshold for *P* < 5 × 10^−8^. **b** and **c**: the regional plots for the two significant loci plotted using LocusZoom. The lead SNPs and functional SNPs after imputation are marked in the plot. **b** the locus located in 4q23 region with 230 SNPs with *P* < 5 × 10^−8^. The top index SNP was rs2075633 on *ADH* (*P* = 6.375 × 10^−15^). The nonsynonymous SNP rs1229984 was also present in this locus (*P* = 1.03 × 10^−12^). **c** the significant locus located in 12q24.12-12q24.13, with 5 SNPs with *P* < 5 × 10^−8^. The top index SNP was *BRAP* rs11066001 (*P* = 1.28 × 10^−8^) and the nonsynonymous SNP was *ALDH2* rs671 (*P* = 3.98 × 10^−8^)

Demographics for the discovery cohort (653 cases and 2854 controls) are summarized in Table 1. Our analysis identified two significant loci. The Manhattan plot is shown in Fig. 1a. The first locus mapped to the 4q23 region, and included 230 SNPs with *P* < 5 × 10^−8^ (regional plot is shown in Fig. 1b). The top index SNP was rs2075633 on *ADH1B* (*P* = 6.64 × 10^−16^). Annovar annotation showed that among these SNPs, there were 14 SNPs annotated as exonic (2 SNPs), ncRNA_exonic (1 SNP), upstream (5 SNPs), downstream (3 SNPs), and 3′UTR (3 SNPs). The well-known nonsynonymous SNP rs1229984, which leads to an amino acid change *(ADH1B*: H48R*, ADH1B*: H8R) and is known to be functional based on biochemistry studies, was present in this locus (*P* = 3.93 × 10^−13^)^37^.

**Table 1:** Characteristics for the discovery and replication cohorts

	*N* (male/female)	Age (SD)	Education (years)	FNTD (SD)	Onset age of regular drinking (SD)
Discovery					
Alcoholism	653 (653/0)	44.81 (9.12)	10.46 (2.79)	5.50 (2.35)	22.67 (7.09)
Control	2854 (1842/1012)	34.12 (6.42)	–	–	–
Replication					
Alcoholism	146 (146/0)	41.79 (8.90)	11.07 (3.19)	4.56 (2.66)	17.41 (4.28)
Control	200 (200/0)	29.21 (9.45)	–	–	–

*SD* standard deviation, *FNTD* the Fagerstrom test for nicotine dependence

The second significant locus was located at 12q24.12-12q24.13 (Fig. 1c). There were 5 SNPs with *P* < 5 × 10^−8^ in this locus. The top index SNP was *BRAP* rs11066001 (*P* = 1.63 × 10^−9^). Annovar annotation showed 3 SNPs as exonic or ncRNA_exonic, in which, *ALDH2* rs671 (*P* = 3.44 × 10^−9^) is a well-known nonsynonymous SNP leading to amino acid change (ALDH2: E504K) and also known to be functional^38^.

Since our patients were all male, we included only male controls (*n* = 1842) for the association analysis. Association analysis using only male controls resulted in the same two significant loci derived using all controls. However, the top lead SNPs (rs13106840 for chr4 locus, rs78069066 for chr12 locus) (Fig. S1) differed. The two lead SNPs (rs2075633 and rs11066001) and two functional SNPs (rs1229984 and rs671) from association analysis using all HCs were also significant using male only controls. Details are presented in Table 2.

**Table 2:** The top SNPs for alcohol case-control GWAS

SNP	Chr	Pos (hg19)	A1	A2	Gene	Discovery stage								Replication stage				Meta-analysis
						Cases vs. all controls				Cases vs. male controls								
						MAF^a^	OR	SE	*P*	MAF^a^	OR	SE	*P*	MAF^a^	OR	SE	*P*	*P*
rs2075633	4	100238998	C	T	*ADH1B*	0.43/0.23	0.397	0.070	6.37 × 10^−15^	0.43/0.24	0.411	0.073	1.22 × 10^−17^	0.59/0.25	0.224	0.229	5.88 × 10^−11^	4.79 × 10^−21^
rs1229984	4	100239319	C	T	*ADH1B*	0.51/0.31	2.313	0.068	1.03 × 10^−12^	0.49/0.30	2.250	0.071	1.13 × 10^−15^	0.23/0.70	6.106	0.229	2.42 × 10^−15^	3.59 × 10^−20^
rs11066001	12	112119171	C	T	*BRAP*	0.05/0.19	0.210	0.149	1.28 × 10^−8^	0.05/0.19	0.246	0.142	1.72 × 10^−9^	0.04/0.21	0.158	0.392	2.42 × 10^−6^	7.15 × 10^−12^
rs671	12	112241766	A	G	*ALDH2*	0.05/0.19	0.202	0.152	3.98 × 10^−8^	0.05/0.18	0.219	0.151	1.38 × 10^−9^	0.06/0.21	0.196	0.350	3.06 × 10^−6^	2.91 × 10^−11^

*OR* odds ratio, *SE* standard error, which was calculated as (logOR-log(OR_lower))/1.96

^a^MAF in cases/MAF in controls

### Genetic association replication

We genotyped two lead SNPs (rs2075633 and rs11066001) and two functional SNPs implicated in numerous prior GWAS (rs1229984 and rs671) for the two significant loci Chr 4 and Chr12 for replication using 146 patients and 200 controls. The promising SNPs had significantly different distribution in case vs. controls in the replication cohort (*P* = 5.88 × 10^−11^ for rs2075633, *P* = 2.42 × 10^−15^ for rs1229984, *P* = 2.42 × 10^−6^ for rs11066001, and *P* = 3.06 × 10^−6^ for rs671), as well as in the meta-analysis cohort (*P* = 4.80 × 10^−21^, 3.59 × 10^−20^, 7.15 × 10^−12^, 2.91 × 10^−11^, respectively) (Table 2).

### Genetic association with MAST, BIS, and alcohol use characteristic features

We assessed the genetic effects of the two functional SNPs (rs1229984 and rs671) on AD related phenotypes. After quality control, the detailed sample size for each genetic-phenotype analysis is shown in Table 3. After Bonferroni correction, only rs1229984 at *ADH1B* was associated with MAST symptom counts (*P* = 6.75 × 10^−5^), BIS-assessed total score (5.93 × 10^−3^), and usual drink volume per day (*P* = 4.34 × 10^−5^). While rs671 on *ALDH2* showed no significant association with alcohol related phenotypes after Bonferroni correction (Table 3). We further verified the association of rs1229984 with BIS subscales (attentional, motor, and non-planning). Only non-planning showed uncorrected nominal significance (*P* = 0.022), while attentional and motor was not significant (*P* = 0.222 and 0.287, respectively).

**Table 3:** Association between the two functional AD SNPs with AD characteristics and BIS in the discovery cohort

phenotype	samples	mean (SD)	CHR	SNP	A1	BETA	SE	*P*
MAST	435	10.25 (5.11)	4	rs1229984	C	1.514	0.3743	**6.75** **×** **10**^−**5**^
			12	rs671	A	−1.776	0.9548	0.064
BIS Sum	426	34.36 (19.81)	4	rs1229984	C	3.956	1.427	**5.93** **×** **10**^−**3**^
			12	rs671	A	6.765	3.627	0.063
Age onset of regular drink (year)	409	22.72 (7.03)	4	rs1229984	C	−0.9016	0.544	0.0986
			12	rs671	A	2.443	1.307	0.06254
Duration of AD (year)	400	22.85 (10.25)	4	rs1229984	C	0.7295	0.524	0.165
			12	rs671	A	−2.542	1.261	0.04458
Usual daily drink (standard drinks)	380	7.69 (6.91)	4	rs1229984	C	2.087	0.5012	**4.34** **×** **10**^−**5**^
			12	rs671	A	−0.8712	1.534	0.5704
Maximum daily drinking (standard drinks)	208	10.10 (8.81)	4	rs1229984	C	0.9966	0.9762	0.3092
			12	rs671	A	1.538	2.525	0.5434

*MAST* the Michigan Alcoholism Screening Test, *BIS* Barratt Impulsiveness Scale 11th version, *SD* standard deviation, *SE* standard error

To adjust for multiple testing, we applied the Bonferroni correction *P* value < 0.0083 (*p* < 0.05/6)

To further understand the association between rs1229984, BIS, and AD severity (MAST assessed), we performed mediation analysis. As shown in Fig. S2A, the total effects and direct effect of rs1229984 on AD severity and the indirect effect of rs1229984 on AD severity through the mediation of BIS_total score were all significant (*P* = 0.0001, 0.0009, 0.013 separately). In the other direction, the total effect of rs1229984 on impulsivity and the indirect of rs1229984 on impulsivity through the mediation of AD severity were significant (Fig. S2B, *P* = 0.0045, 0.0013, separately).

### Gene and gene-set association analysis

We identified 14 genes with significance levels (*P* < 0.05/18227 = 2.74 × 10^−6^). These genes were located in our two significant loci on Chr4 and Chr12 (Table S1). Further gene-set association analysis identified 8 GO gene sets with significance levels (*P* < 0.05/6925 = 7.22 × 10^−6^). The most significant gene-sets were related to ethanol metabolism (i.e.,“ethanol_oxidation”, “alcohol_dehydrogenase_activity, zinc-dependent”, “aldehyde_dehydrogenase_activity”) (Table S2).

### Genetic overlap of AD risk across different ethnic populations

**Fig. 2:**
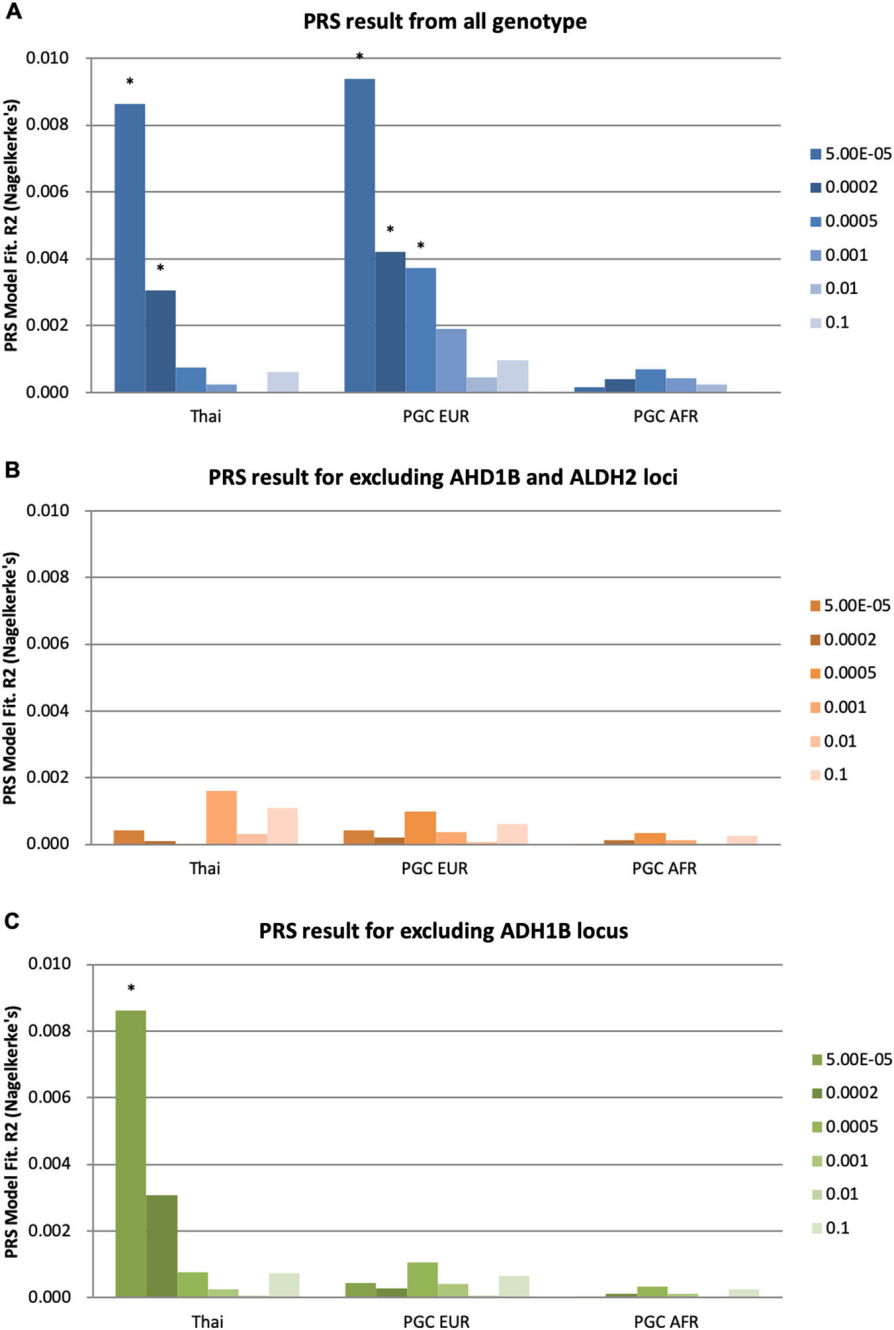
Predicting alcohol dependence in Han Chinese using the polygenic risk score (PRS) for alcohol dependence (AD) in different populations. **a** was the result of the primary PRS analysis; **b** was the result after removing the SNPs within the ±2 MB region of the top two loci; **c** was the result after removing the SNPs within the ±2 MB region of chr4 *ADH* locus. Thai denotes the Thai cohort, PGC EA for European Americans, PGC AA for African Americans. Asterisks (*) denotes *P* < 0.05/6 = 0.0083

PRS analysis at different *P*-value thresholds (5.00 × 10^−5^/0.0002/0.0005/0.001/0.01/0.1) was used to compare the genetic overlap of AD across different ethnic populations. The results from the best *P* value threshold showed that the PRS from Thai AD cohorts, and AD in PGC EA were significantly associated with AD in Han Chinese populations (*P* = 1.03 × 10^−6^ and *P* = 2.83 × 10^−7^, respectively). However, PRS from AD in the AA population were not associated with AD in Han Chinese population after multiple correction (Fig. 2a and Table S3).

We checked whether the association of PRS was mainly driven by the significant loci. We firstly removed all SNPs within the upstream 2 MB and downstream 2 MB region of the two lead SNPs from the genotype data, and then performed PRS. As shown in Fig. 2b and Table S3, the results showed, after removing the top two loci, the PRS from Thai, EA and AA were not associated with AD. Since the rs671 locus only exists in Asian population, we also tried to only remove the SNPs around the 2MB of the lead SNP of chr4 *ADH1B* locus, the result showed the PRS from EA and AA were not significant, while the PRS from Thai was still significant. The result showed the original PRS from Thai was mainly from the signal of the top two loci, while the original PRS from EA was mainly from the chr4 *ADH1B* locus.

## Discussion

We are the first to perform a case-control AD GWAS in Han Chinese. Our findings demonstrated that the genetic variants in tight LD with *ADH1B* rs1229984 and *ALDH2* rs671 were the leading genetic factors for AD in Han Chinese. The genetic associations were replicated in an additional case-control cohort which substantiated the genetic effects on AD severity and BIS-assessed impulsivity total score. Cross-ethnic analysis suggested the similar genetic distribution of rs1229984*ADH1B and 671*ALDH2 loci between Han Chinese and Thai; while similar rs1229984*ADH1B distribution between Han Chinese and European ethnic populations.

*ADH1B* rs1229984 is a well-known coding SNP in the 4q23 region associated with alcohol abuse. The T allele frequency is high in Asian (T = 0.697, 1000 Genomes) but rare in most European (T = 0.029) and African populations (T = 0.002)^39^. The protective T allele (ADH1B*47His) is more frequent in normal controls compared to AD patients. This protective allele results in an enhanced catalytic activity to increase blood levels and flushing. *ADH1B* rs1229984 has a significant effect size for genetic association with AD across deferent population groups (OR = 2.06/1.10/2.24 in European/African/Asian separately)^9,11,40^. Our results remarkably replicated this well-studied locus of AD in Han Chinese (OR = 2.313) and was concordant with the findings from the largest GWAS meta-analysis of DSM-IV diagnosed AD to date^40^.

Several studies have provided evidence for the association of rs1229984 with alcohol related phenotypes^8,41–43^, including AD symptom count, maximum drink volume, flushing and even pub attendance. Consistent with these findings, we also observed strong effects of rs1229984 on AD symptom counts, BIS_total score and usual daily drink in Chinese AD patients. Elevated impulsivity is a well-established behavioral trait in AD subjects^44^, but the direction between AD and impulsivity was inconclusive. Our mediation analysis suggested that BIS-assessed impulsivity not only could mediate the vulnerability effect of rs1229984 risk allele for on AD severity, it may also be affected by rs1229984 among AD patients through the mediation of AD severity.

SNP rs671 is a non-synonymous polymorphism in *ALDH2* that is common in certain Asian populations and absent in most other populations. The A-to-G substitution of this functional variant in *ALDH2* leads to a lysine to glutamic substitution. The presence of a single rs671 A allele (ALDH2*2) leads to a drastic decrease in alcohol dehydrogenase activity resulting in protection against heavy drinking by increasing blood acetaldehyde levels resulting in a strong flushing reaction^45^. Although allele frequency is variable across Asia, the frequency of the minor allele A is much higher in some Asian populations (~0.20) compared to European and other populations (<0.01) as derived from the 1000 Genome Project. A meta-analysis of 15 Asian studies found that the rs671 A allele was significantly protective against AD^46^. Previous studies have shown that rs671 was associated with alcohol-related traits, such as 24-h maximum drinks and flushing in Asian populations^7,10,47^. Hence, our result validated the important role of rs671 for alcoholism in Han Chinese. However, we only found nominal significant effects of the rs671 genotype on AD duration, BIS attentional and nonplanning impulsivity (not significant after Bonferroni correction). This may be due to our limited sample size and the strict multiple testing correction. It may also suggest that *ALDH2* rs671 has dichotomic effects (null/effective) on the physical reaction to alcohol, which is less reflected or mediated by the continuous AD phenotypes.

The lead SNPs for both these two significant loci were not the well-implicated functional SNPs. The lead SNP rs2075633 at 4q23 locus was adjacent to rs1229984 (distance = 321 bp, LD *r*^2^ = 0.727). The lead SNP rs11066001 and functional SNP rs671 in the 12q24.12-12q24.13 locus was more distant (122,595 bp) but had strong LD (*r*^2^ = 0.982). This inconsistency may be due to the lack of precision in mapping the location in our small-cohort GWAS^48^. We need to reconsider these results by integrating functional information from previous studies. However, consistent with previous studies^7,9,10,15,47,49^, our findings support *ADH1B* rs1229984 and *ALDH2* rs671 as being important genetic factors for the risk of AD and alcohol consumption in Asian populations. We could not exclude the potential function of other significant and indicative SNPs from our results. Similar results have been observed in pervious small GWAS^7^.

Since diverging from the ancestral African population, Asians have acquired *ADH* variants. The inactive *ALDH* variant could protect against heavy drinking and AD^50^. Differences and similarities in alcohol-related genetic factors among various populations have been reported in several studies^47,51^. Previous studies have found that the genetic distribution of several key alcohol-related polymorphisms in Han Chinese differ greatly from Europeans and Africans but are similar among Asian populations^52^. Our results suggest that the overlap genetic risk factors of AD between Han Chinese with Thai and EA was entirely drived by the *ADH* and *ALDH2* loci. AD in Chinese has similar genetic distribution of rs1229984*ADH1B and 671*ALDH2 loci with Thai, has similar rs1229984*ADH1B distribution with European ethnic populations.

Sample size was a major limitation in our study, notwithstanding the strong genome-wide significant results obtained. To identify novel loci that go beyond pharmacokinetic mechanism and explore the association of these loci with alcohol related phenotypes, studies using larger sample cohorts and meta-analyses of existing cohorts should be performed. The effect of rare variants^53^ and genetic interactions^54^ could also impact AD, but were not considered in this study. Furthermore, most alcoholics in China do not seek treatment, hence we could not rule out potential interference (e.g., personality, economic level, severity degree and so on) between patients seeking treatment and those who did not.

In conclusion, we found and validated two genome-wide significant loci that were highly linked to either *ADH1B* rs1229984 or *ALDH2* for AD in Han Chinese. The genetic association between rs1229984 and AD was substantiated by its effects on AD symptom scores, daily drink volume and BIS_total scores in Han Chinese. In addition, AD in Chinese has similar genetic distribution of rs1229984*ADH1B and 671*ALDH2 loci with Thai, and has similar rs1229984*ADH1B distribution with European ethnic populations. These findings will help improve our understanding of the mechanism of AD and may perhaps facilitate the development of ethnic-specific prevention and treatment strategies for AD in Han Chinese.

## Supplementary information

supplemental materials
