# Supplementary material for: Genome-wide association study of alcohol dependence in male Han Chinese and cross-ethnic polygenic risk score comparison
Source: Transl Psychiatry. 2019 Oct 7;9:249. doi: 10.1038/s41398-019-0586-3 (PMC6779867; doi:10.1038/s41398-019-0586-3)
Supplement: Supplementary file 1 — supplemental materials [file 41398_2019_586_MOESM1_ESM.docx]

**Supplementary Materials**

**Genome-wide association study of alcohol dependence in male Han Chinese and** **cross-ethnic polygenic risk score comparison**

Yan Sun^1#^, Suhua Chang^2#^, Fan Wang^3,4^, Hongqiang Sun^2^, Zhaojun Ni^2^, Weihua Yue^2^, Hang Zhou^5,6^, Joel Gelernter^5,6^, Robert T. Malison^5,7^, Rasmon Kalayasiri^8,9^, Lin Lu^1.2^*, Jie Shi^1,10,11,12^*

*1 National Institute on Drug Dependence, Peking University, Beijing, 100191, China*

*2 Peking University Sixth Hospital, Peking University Institute of Mental Health, NHC Key Laboratory of Mental Health (Peking University), National Clinical Research Center for Mental Disorders (Peking University Sixth Hospital), Peking University, Beijing 100191, China*

*3 Beijing Hui Long Guan Hospital, Beijing, 100096, China*

*4* *The Second Affiliated Hospital, Xinjiang Medical University,* *Urumqi, 830063, China*

*5* *Department of Psychiatry, Yale University School of Medicine,* *New Haven, CT 06511, USA*

*6* *VA Connecticut Healthcare System, West Haven, CT 06516, USA*

*7* *Clinical Neuroscience Research Unit,* *Connecticut Mental Health Center, New Haven, CT 06519, USA*

*8* *Department of Psychiatry, King Chulalongkorn Memorial Hospital,* *Bangkok, 10330, Thailand*

*9 Department of Psychiatry, Faculty of Medicine,* *Chulalongkorn University, 10330, Bangkok 10330, Thailand*

*10 Beijing Key Laboratory of Drug Dependence Research, Peking University, Beijing, 100191, China*

*11 The State Key Laboratory of Natural and Biomimetic Drugs, Peking University, Beijing, 100191, Beijing, China;*

*12* *The Key Laboratory for Neuroscience of the Ministry of Education and Health, Peking University, Beijing 100191, China*

^#^Equally contributed to this work

*Correspondence:

Prof. Jie Shi (shijie@bjmu.edu.cn)

National Institute on Drug Dependence, Peking University, 38 Xueyuan Road, Haidian District, Beijing 100191, China. Tel: 86-10-8280-1593, Fax: 86-10-62032624

Or

Prof. Lin Lu (linlu@bjmu.edu.cn)

Institute of Mental Health, Peking University, 51 Huayuan Bei Road, Beijing 100191, China, Tel: +86-10-82805308; Fax: +86-10-62032624

File contents include;

Supplemental figure S1-S2

Supplemental table S1-S3

**Figure S1** Manhattan plot of the association analysis for the discovery stage of alcohol GWAS using male only controls (Red line denotes the threshold of *P*<5× 10^-8^).

**
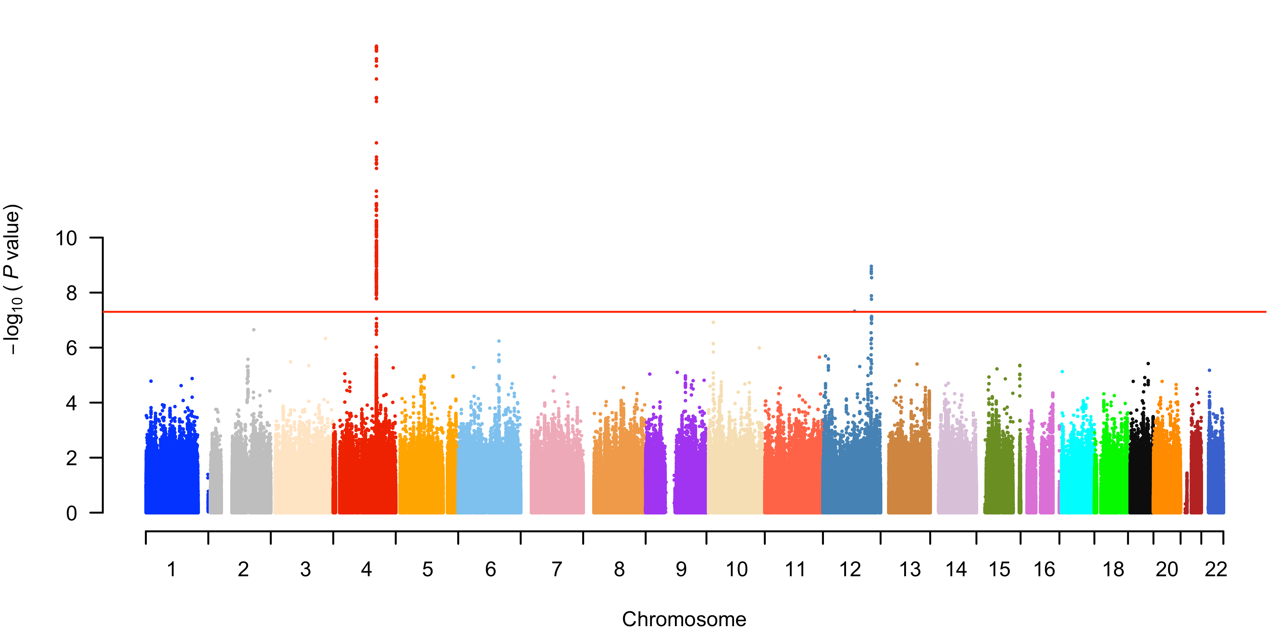
**

**Figure S2** Mediation analysis result for significant SNP (rs1229984), impulsivity (BIS-11 sum) and AD severity (MAST).

Mediation analysis was performed using the model 4 in PROCESS. The model 4 included three variables, X (independent variable), Y (outcome variable), M (mediation variable), and four regressions, including X variable predict M (path a), M predict Y (path b), X variable predict Y (path c, total effect), and the direct effect of X on Y. So the indirect effect of X on Y through M equals c-c’.

**
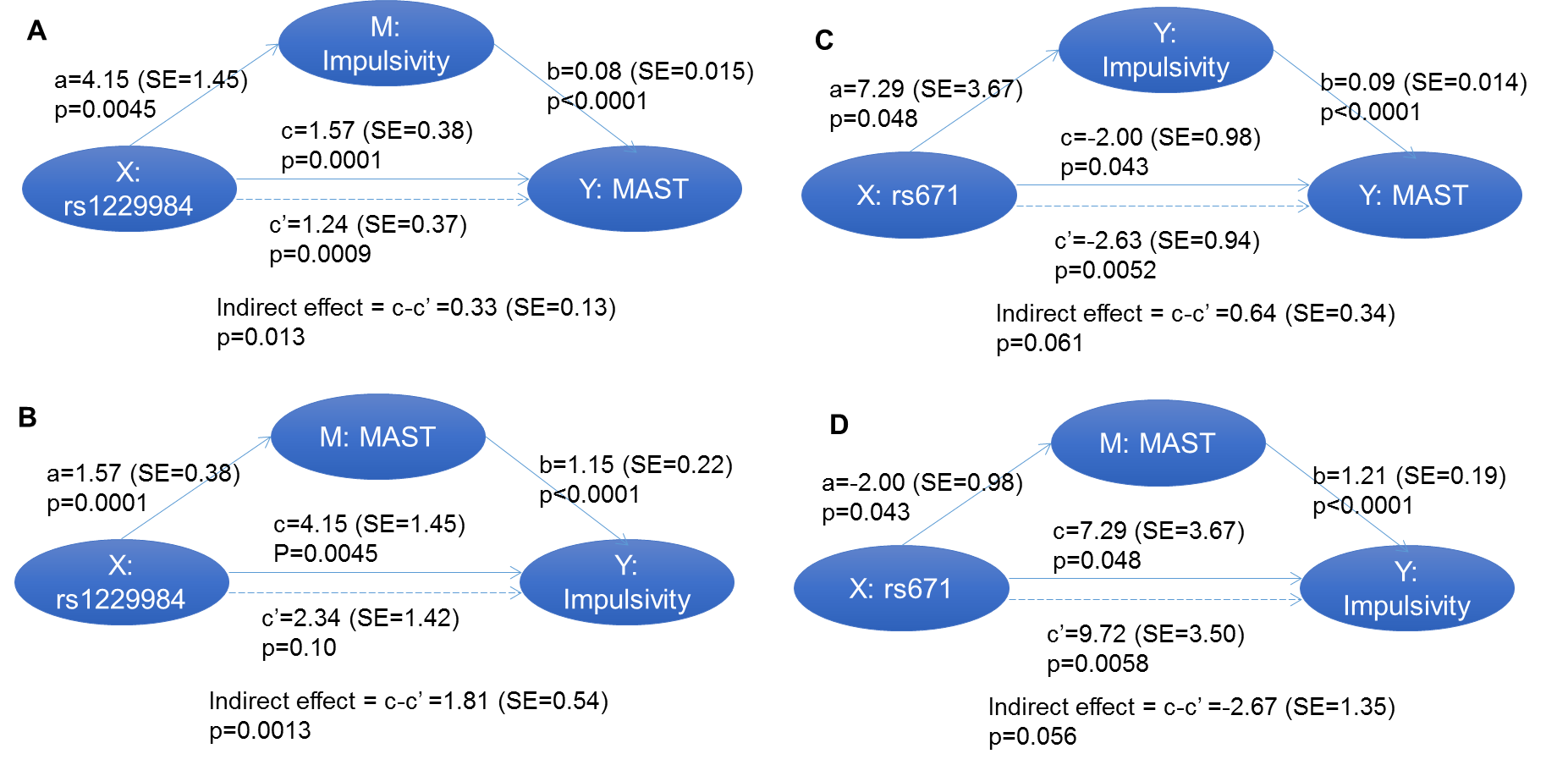
**

**Table S1** Gene association analysis from MAGMA for AD GWAS. Genes that passed the Bonferroni correction (*P*<0.05/18227=2.74×10^-6^) are shown.

| **CHR** | **START** | **STOP** | **NSNPS** | **NPARAM** | **ZSTAT** | **P** | **Gene Symbol** | **Gene Name** |
| --- | --- | --- | --- | --- | --- | --- | --- | --- |
| 4 | 100192523 | 100217185 | 47 | 6 | 10.656 | 8.21×10^-27^ | ADH1A | alcohol dehydrogenase 1A (class I), alpha polypeptide |
| 4 | 100118795 | 100145403 | 34 | 6 | 9.021 | 9.37×10^-20^ | ADH6 | alcohol dehydrogenase 6 (class V) |
| 4 | 100222527 | 100247572 | 47 | 7 | 8.874 | 3.54×10^-19^ | ADH1B | alcohol dehydrogenase 1B (class I), beta polypeptide |
| 12 | 112074950 | 112128790 | 37 | 11 | 7.873 | 1.74×10^-15^ | BRAP | BRCA1 associated protein |
| 4 | 100252649 | 100278917 | 150 | 7 | 7.859 | 1.94×10^-15^ | ADH1C | alcohol dehydrogenase 1C (class I), gamma polypeptide |
| 12 | 112459493 | 112551826 | 113 | 14 | 7.742 | 4.89×10^-15^ | NAA25 | N(alpha)-acetyltransferase 25, NatB auxiliary subunit |
| 12 | 112592992 | 112825113 | 306 | 19 | 7.534 | 2.46×10^-14^ | HECTD4 | HECT domain E3 ubiquitin protein ligase 4 |
| 12 | 112118857 | 112199911 | 93 | 18 | 7.502 | 3.13×10^-14^ | ACAD10 | acyl-CoA dehydrogenase family member 10 |
| 4 | 100039832 | 100070449 | 28 | 4 | 7.442 | 4.98×10^-14^ | ADH4 | alcohol dehydrogenase 4 (class II), pi polypeptide |
| 12 | 112199691 | 112252789 | 60 | 13 | 7.417 | 6.00×10^-14^ | ALDH2 | aldehyde dehydrogenase 2 family member |
| 12 | 112558349 | 112596408 | 41 | 8 | 7.245 | 2.16×10^-13^ | TRAFD1 | TRAF-type zinc finger domain containing 1 |
| 4 | 99987129 | 100014931 | 20 | 5 | 7.044 | 9.33×10^-13^ | ADH5 | alcohol dehydrogenase 5 (class III), chi polypeptide |
| 4 | 100328418 | 100361667 | 70 | 14 | 6.216 | 2.55×10^-10^ | ADH7 | alcohol dehydrogenase 7 (class IV), mu or sigma polypeptide |
| 12 | 111466828 | 111793358 | 263 | 35 | 4.988 | 3.05×10^-7^ | CUX2 | cut like homeobox 2 |
| 12 | 111343623 | 111363404 | 36 | 3 | 4.689 | 1.37×10^-6^ | MYL2 | myosin light chain 2 |
| 4 | 99911788 | 99988960 | 80 | 11 | 4.613 | 1.99×10^-6^ | METAP1 | methionyl aminopeptidase 1 |

**Table S2** Gene-set enrichment analysis from MAGMA for AD GWAS using GO as gene set database. Gene-sets that passed the Bonferroni correction (*P*<0.05/6925=7.22×10^-6^) are shown.

| **VARIABLE** | **NGENES** | **BETA** | **BETA_STD** | **SE** | ***P*** | **GO Name** |
| --- | --- | --- | --- | --- | --- | --- |
| GO:0006069 | 11 | 2.01 | 0.0494 | 0.293 | 3.59×10^-12^ | ethanol oxidation |
| GO:0004024 | 4 | 2.44 | 0.0361 | 0.506 | 7.65×10^-7^ | alcohol dehydrogenase activity, zinc-dependent |
| GO:0004029 | 9 | 1.37 | 0.0305 | 0.287 | 8.61×10^-7^ | aldehyde dehydrogenase (NAD) activity |
| GO:0030663 | 2 | 2.53 | 0.0265 | 0.530 | 9.44×10^-7^ | COPI coated vesicle membrane |
| GO:0019228 | 14 | 1.06 | 0.0293 | 0.226 | 1.45×10^-6^ | regulation of action potential in neuron |
| GO:0060765 | 5 | 1.58 | 0.0262 | 0.349 | 3.01×10^-6^ | regulation of androgen receptor signaling pathway |
| GO:0051583 | 2 | 1.88 | 0.0197 | 0.422 | 4.15×10^-6^ | dopamine uptake |
| GO:0071398 | 3 | 2.04 | 0.0262 | 0.463 | 5.33×10^-6^ | cellular response to fatty acid |

**Table S3** Polygenic risk score analysis for each *P*-value threshold. *P*-value < 0.00833 (0.05/6) are shown in bold. Group “All” denotes the primary PRS analysis, “Excluded” denotes excluding the SNPs around the 2MB region of the top two loci, “ExcludedChr4” denotes excluding the SNPs around the 2MB region of the chr4 ADH locus.

| **Threshold** | **Group** | **Base** | **R2** | **P** | **Coefficient** | **Standard Error** | **Num SNP** |
| --- | --- | --- | --- | --- | --- | --- | --- |
| 5.00E-05 | All | PGC AFR | 1.7270E-04 | 0.483 | -2.8175 | 4.0156 | 97 |
|  |  | PGC EUR | 9.3961E-03 | **2.828E-07** | 26.6485 | 5.1901 | 61 |
|  |  | Thai | 8.6299E-03 | **1.034E-06** | -4.3021 | 0.8807 | 27 |
|  | Excluded | PGC AFR | 1.0777E-05 | 0.859 | 0.7082 | 4.0005 | 93 |
|  |  | PGC EUR | 4.2693E-04 | 0.266 | 6.9568 | 6.2510 | 58 |
|  |  | Thai | 4.1981E-04 | 0.269 | -1.0493 | 0.9496 | 22 |
|  | ExcludedChr4 | PGC AFR | 1.0777E-05 | 0.859 | 0.7082 | 4.0005 | 93 |
|  |  | PGC EUR | 4.2693E-04 | 0.266 | 6.9568 | 6.2510 | 58 |
|  |  | Thai | 8.6299E-03 | **1.034E-06** | -4.3021 | 0.8807 | 27 |
| 0.0002 | All | PGC AFR | 3.9342E-04 | 0.290 | -7.9162 | 7.4782 | 343 |
|  |  | PGC EUR | 4.2075E-03 | **5.658E-04** | 44.8101 | 12.9978 | 255 |
|  |  | Thai | 3.0628E-03 | **2.976E-03** | -5.8418 | 1.9668 | 105 |
|  | Excluded | PGC AFR | 1.1042E-04 | 0.571 | -4.2369 | 7.4774 | 336 |
|  |  | PGC EUR | 2.1340E-04 | 0.431 | 11.0912 | 14.0834 | 248 |
|  |  | Thai | 9.0234E-05 | 0.609 | -1.0601 | 2.0696 | 100 |
|  | ExcludedChr4 | PGC AFR | 1.1042E-04 | 0.571 | -4.2369 | 7.4774 | 336 |
|  |  | PGC EUR | 2.8392E-04 | 0.364 | 12.8347 | 14.1308 | 250 |
|  |  | Thai | 3.0628E-03 | 0.003 | -5.8418 | 1.9668 | 105 |
| 0.0005 | All | PGC AFR | 6.8833E-04 | 0.162 | -17.0593 | 12.1987 | 773 |
|  |  | PGC EUR | 3.7244E-03 | 0.001 | 63.3151 | 19.5175 | 566 |
|  |  | Thai | 7.5897E-04 | 0.138 | -5.3974 | 3.6368 | 285 |
|  | Excluded | PGC AFR | 3.3568E-04 | 0.324 | -12.1200 | 12.2798 | 766 |
|  |  | PGC EUR | 9.9278E-04 | 0.090 | 35.2270 | 20.7663 | 558 |
|  |  | Thai | 2.3575E-05 | 0.793 | 0.9845 | 3.7603 | 280 |
|  | ExcludedChr4 | PGC AFR | 3.3568E-04 | 0.324 | -12.1200 | 12.2798 | 766 |
|  |  | PGC EUR | 1.0474E-03 | 0.082 | 36.2578 | 20.8158 | 561 |
|  |  | Thai | 7.5897E-04 | 0.138 | -5.3974 | 3.6368 | 285 |
| 0.001 | All | PGC AFR | 4.3604E-04 | 0.265 | -19.9054 | 17.8657 | 1482 |
|  |  | PGC EUR | 1.8996E-03 | 0.020 | 65.3048 | 28.1482 | 1018 |
|  |  | Thai | 2.4148E-04 | 0.402 | 4.5836 | 5.4709 | 593 |
|  | Excluded | PGC AFR | 1.1597E-04 | 0.562 | -10.5876 | 18.2376 | 1473 |
|  |  | PGC EUR | 3.7235E-04 | 0.298 | 30.6042 | 29.4367 | 1009 |
|  |  | Thai | 1.6055E-03 | 0.031 | 12.1175 | 5.6186 | 588 |
|  | ExcludedChr4 | PGC AFR | 1.1597E-04 | 0.562 | -10.5876 | 18.2376 | 1473 |
|  |  | PGC EUR | 4.0073E-04 | 0.281 | 31.7823 | 29.4713 | 1012 |
|  |  | Thai | 2.4148E-04 | 0.402 | 4.5836 | 5.4709 | 593 |
| 0.01 | All | PGC AFR | 2.4990E-04 | 0.399 | -47.3225 | 56.0911 | 10705 |
|  |  | PGC EUR | 4.5759E-04 | 0.253 | 104.4670 | 91.4639 | 7022 |
|  |  | Thai | 2.0518E-05 | 0.807 | 4.8428 | 19.8251 | 4933 |
|  | Excluded | PGC AFR | 9.2391E-06 | 0.870 | -9.4006 | 57.3536 | 10669 |
|  |  | PGC EUR | 6.0949E-05 | 0.674 | 39.9665 | 94.9245 | 7002 |
|  |  | Thai | 3.1443E-04 | 0.339 | 19.0199 | 19.8960 | 4909 |
|  | ExcludedChr4 | PGC AFR | 1.1553E-05 | 0.855 | -10.5124 | 57.3568 | 10680 |
|  |  | PGC EUR | 7.0382E-05 | 0.651 | 42.9491 | 94.9272 | 7011 |
|  |  | Thai | 5.4278E-05 | 0.691 | 7.8602 | 19.7836 | 4923 |
| 0.1 | All | PGC AFR | 1.0396E-06 | 0.957 | 8.5415 | 156.8850 | 49557 |
|  |  | PGC EUR | 9.5754E-04 | 0.099 | 438.7140 | 265.9380 | 37413 |
|  |  | Thai | 6.1566E-04 | 0.181 | 85.8118 | 64.1590 | 30455 |
|  | Excluded | PGC AFR | 2.6433E-04 | 0.381 | 140.9460 | 160.7840 | 49452 |
|  |  | PGC EUR | 6.0387E-04 | 0.186 | 360.8040 | 272.6290 | 37346 |
|  |  | Thai | 1.0831E-03 | 0.076 | 114.3520 | 64.4940 | 30387 |
|  | ExcludedChr4 | PGC AFR | 2.4647E-04 | 0.397 | 135.8850 | 160.5250 | 49496 |
|  |  | PGC EUR | 6.5662E-04 | 0.168 | 376.0020 | 272.4880 | 37377 |
|  |  | Thai | 7.2480E-04 | 0.147 | 93.1726 | 64.2091 | 30423 |
